# Supplementary material for: Local PI(4,5)P2 synthesis by septin-associated PIPKIγ isoforms controls centralspindlin association with the midbody during cytokinesis
Source: Nat Commun. 2026 Feb 7;17:1482. doi: 10.1038/s41467-026-69224-3 (PMC12886786; doi:10.1038/s41467-026-69224-3)
Supplement: Supplementary file 1 — Supplementary Information [file 41467_2026_69224_MOESM1_ESM.pdf]

Supplementary Information for

Local PI(4,5)P_2 synthesis by septin-associated PIPKIγ isoforms controls centralspindlin association with the midbody during cytokinesis

Giulia Russo[1], Nadja Hümpfer[2], Nina Jaensch[3], Steffen Restel[1], Christopher Schmied[1], Florian Heyd[3], Martin Lehmann[1], Helge Ewers[2], Volker Haucke[1,2,4*] & Michael Krauss[1,5*]

*Correspondence to: krauss@fmp-berlin.de, or haucke@fmp-berlin.de

This PDF includes:

Supplementary Tables 1-3

Supplementary Figures 1-5

Antibodies used in this study

Supplementary Table 1: Primary antibodies

Antibody	Source	Identifier	IF	U-exM	WB
Acetylated tubulin (mouse)	Sigma-Aldrich	T7451	1:2000		
α-tubulin (mouse)	Sigma-Aldrich	T5168		1:400	1:2000
β-tubulin (mouse)	Sigma-Aldrich	T5293		1:400	
PIPKIα (mouse)	Santa-Cruz	sc-398687			1:100
PIPKIβ (mouse)	Santa-Cruz	sc-514169			1:100
PIPKIγ (rabbit)	Home-made in this study				1:500
SEPT2 (rabbit)	Sigma-Aldrich	HPA018481	1:200	1:200	1:500
SEPT5 (mouse)	Santa-Cruz	sc-20040			1:100
SEPT6 (rabbit)	Home-made in this study		1:70		1:250
SEPT7 (rabbit)	Santa-Cruz	sc-20620	1:100		1:500
SEPT7 (rabbit)	TECAN	JP18991	1:250	1:200	
SEPT3 (mouse)	Sigma-Aldrich	WH0055964M3			1:1000
SEPT9 (rabbit)	(Diesenberg et al., 2015)		1:400		
SEPT9 (mouse)	abnova	H0001 1081-M01			1:500
Talin (mouse)	Sigma-Aldrich	T3287			1:1000
GAPDH (mouse)	Sigma-Aldrich	G8795			1:10000
Anillin (goat)	Abcam	ab5910	1:50		
c-Myc (mouse)	Hybridoma clone obtained from DSHB, purified ourselves	9E10			1:400
RFP (rabbit)	Clontech	632496			1:1000
HA (mouse)	Abcam	ab18181	1:500		
OCRL1 (rabbit)	Cell Signaling	8797			1:500
PRC1 (mouse)	Thermo-Fisher	MA1-846	1:1000		
CIT-K (mouse)	BD Transduction Laboratories	611377	1:300		1:500
CIT-K (rabbit)	Abcam	ab86782	1:100		
MKLP1 (rabbit)	GeneTex	GTX120875	1:250	1:250	1:500
MgcRacGAP (goat)	Abcam	Ab2270		1:200	
MgcRacGAP (rabbit)	Proteintech	13739-1-AP	1:500		1:500
GFP (mouse)	Clontech	632381			1:1000
GFP (rabbit)	Abcam	ab6556	1:1000		
PI(4,5)P_2 (mouse, IgM)	Echelon Biosciences	Z-P045	1:100		
pMyosin LC (ser 19)	Cell signaling	3671	1:50		

Supplementary Table 2: Secondary antibodies

Antibody	Conjugate	Source	Identifier	IF	U-exM	WB
Goat anti rabbit	Alexa Fluor 488	Thermo-Fisher	A-11034	1:200 1:400 (PH-PLC staining)		
Goat anti mouse	Alexa Fluor 568	Thermo-Fisher	A-11004	1:200		
Donkey anti goat	Alexa Fluor 488	Thermo-Fisher	A-11055	1:200	1:250	
Donkey anti rabbit	Alexa Fluor 647	Thermo-Fisher	A-31573	1:200	1:250	
Donkey anti mouse	Alexa Fluor 568	Thermo-Fisher	A-10037	1:200	1:250	
Goat anti mouse	Alexa Fluor 647	Thermo-Fisher	A21236	1:200		
Goat anti mouse (IgM)	Alexa Fluor 568	Thermo-Fisher	A21043	1:500		

Goat anti mouse	HRP	Jackson ImmunoResearch	115-035-003			1:2500
Goat anti rabbit	HRP	Jackson ImmunoResearch	111-035-003			1:2500
Goat anti mouse	IRDye® 800CW	LI-COR	926- 32210			1:5000
Goat anti rabbit	IRDye® 680RD	LI-COR	926-68071			1:5000

Supplementary Table 3: Oligonucleotides

DNA oligonucleotide	Sequence 5´-3´
PIPKIγ EcoRI for	GATCAgaattcATGGAGCTGGAGGTACCGG
PIPKIγ-i1 Ct XhoI rev	GATCActcgagTTAAAAGTAGATGTCGGTGGCG
PIPKIγ- i3 Ct XhoI rev	GATCActcgagTTATGTGTCGCTCTCGCCGTCGG
PIPKIγ-i5 Ct XhoI rev	GATCActcgagTTACCCAAAGCCCTTCTGG
PIPKIγ-i1 Ct NotI rev	GATCAgcggccgcTTAAAAGTAGATGTCGGTGGCG
PIPKIγ-i3 Ct NotI rev	GATCAgcggccgcTTATGTGTCGCTCTCGCCGTC
PIPKIγ-i5 Ct NotI rev	GATCAgcggccgcTTACCCAAAGCCCTTCTGG
PIPKIγ-i3/i5 siRNAres for	TTCACcGAcGGcAGaTACTGGATTTACTCTCCCCGCC
PIPKIγ-i3/i5 siRNAres rev	GGCGGGGAGAGTAAATCCAGTAtCTgCCgTCgGTGAA
PIPKIγ-i3/i5 siRNAres Y646A/W647A for	TTCACcGAcGGcAGagccgcgATTTACTCTCCCCGCC
PIPKIγ-i3/i5 siRNAres Y646A/W647A rev	GGCGGGGAGAGTAAATcgcggctCTgCCgTCgGTGAA
PRC1 EcoRI for	GATCgaattcATGAGGAGAAGTGAGGTGCTG
PRC1 NotI rev	GATCgcggccgcTTAGGACTGGATGTTGGTTGAATT
KI-SEPT6 guide for	CACCGCATCGCTCCTGCGTCCGCCA
KI-SEPT6 guide rev	AAACTGGCGGACGCAGGAGCGATGC
KI-SEPT6 5´HR for	taagctacaacaaggcaaggcttgaccgacGAAGCAGCGTCTCAAGACCTCTAGCC
KI-SEPT6 5´HR rev	gctcacaccggtcatCGCTCCTGCGTCCGCCAGTTCTGCCACGGG
KI-E-SEPT6 eGFP for	gcggacgcaggagcgATGACCGGTGTGAGCAAG
KI-E-SEPT6 eGFP rev	atcggtcgctgccatGGATCCCTTGTACAGCTCG
KI-SEPT6 3´HR for	ctgtacaagggatccATGGCAGCGACCGATATAG
KI-SEPT6 3´HR for	tcgaggctgatcagcgggtttaaacgggccCACAGGTTTTGGCACACAAG

Supplementary Figures

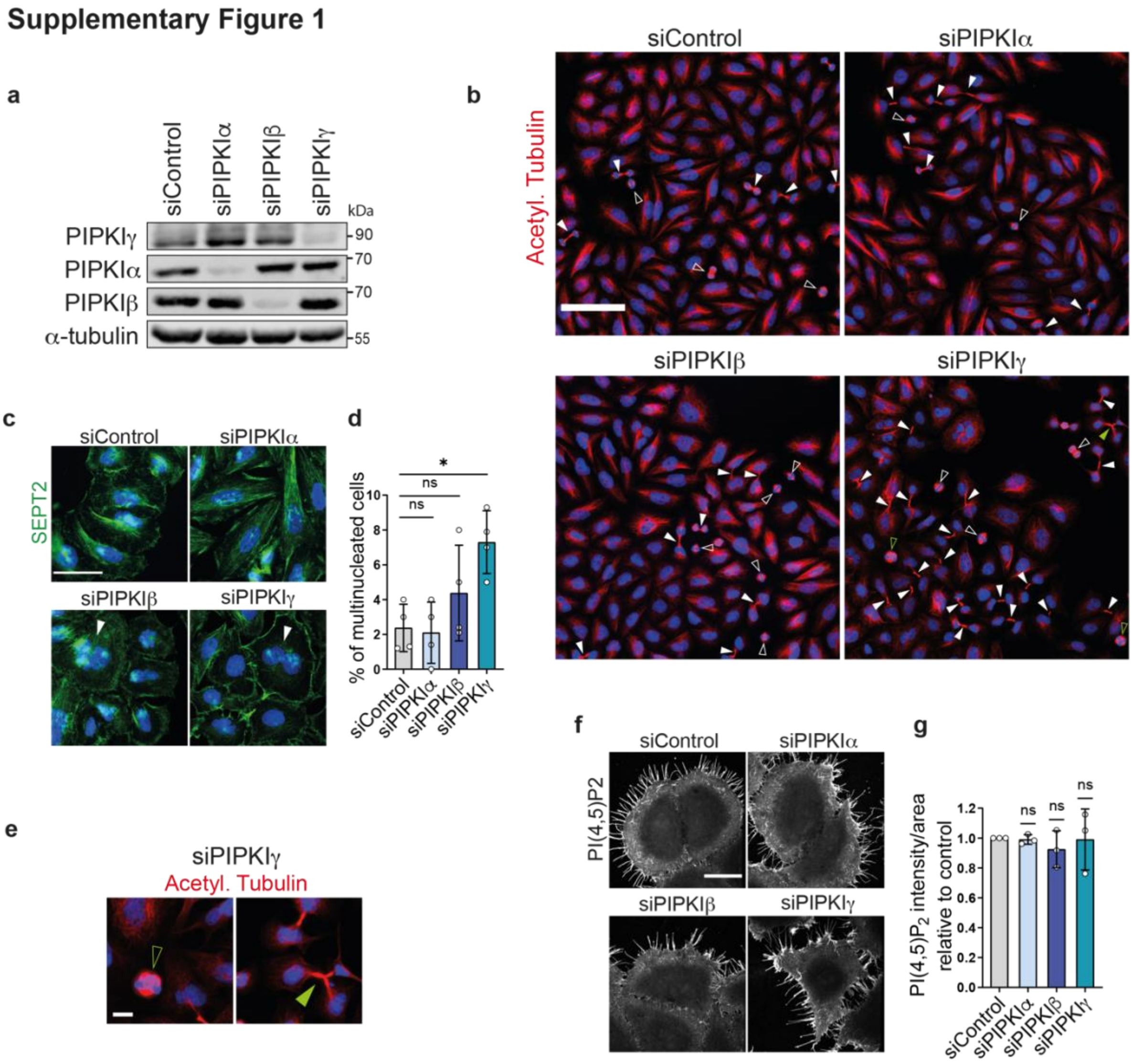

Supplementary Fig. 1 (related to Fig. 1) (a) Western blot analysis show efficient knock-down of PIPKI isozymes. **(b)** HeLa were treated with siRNA control or against PIPKIα, β, γ, and stained for acetylated tubulin and DAPI. Four areas of 1,3 mm^2 were imaged by epi-fluorescence imaging (see Fig.1a-e for quantifications). Representative insets are shown in (e). White arrowheads indicate acetylated tubulin spindles (open), and acetylated tubulin bridges (filled), green arrowheads indicate multipolar spindles (open) and multipolar bridges (filled). Scale bar: 100µm. **(c)** Representative epi-fluorescence images of HeLa cells treated with control siRNA, or with siRNA targeting PIPKIα, β, or γ, and immunostained for SEPT2 and with DAPI, scale bar: 50µm. **(d)** Fraction of multinucleated cells. Data are depicted as mean ± SD (n=4 independent experiments). Statistics: 1-way ANOVA, followed by Dunnett's multiple comparison test. **(e)** Insets from (b) depicting multipolar spindles and multipolar bridges, scale bar: 5µm. **(f)** Representative confocal images of HeLa cells treated with control siRNA, or with siRNA targeting PIPKIα, β, or γ, and stained for PI(4,5)P$_2$ with purified eGFP-tagged PH-PLC δ1 domain, scale bar: 30µm. **(g)** Relative intensities of PI(4,5)P$_2$ per cell area, depicted as mean ± SD (n=3 independent

experiments). Statistics: two-tailed one sample t test. *$P < 0.05$. Source data and *P*-values are provided as a Source Data file.

Supplementary Figure 2

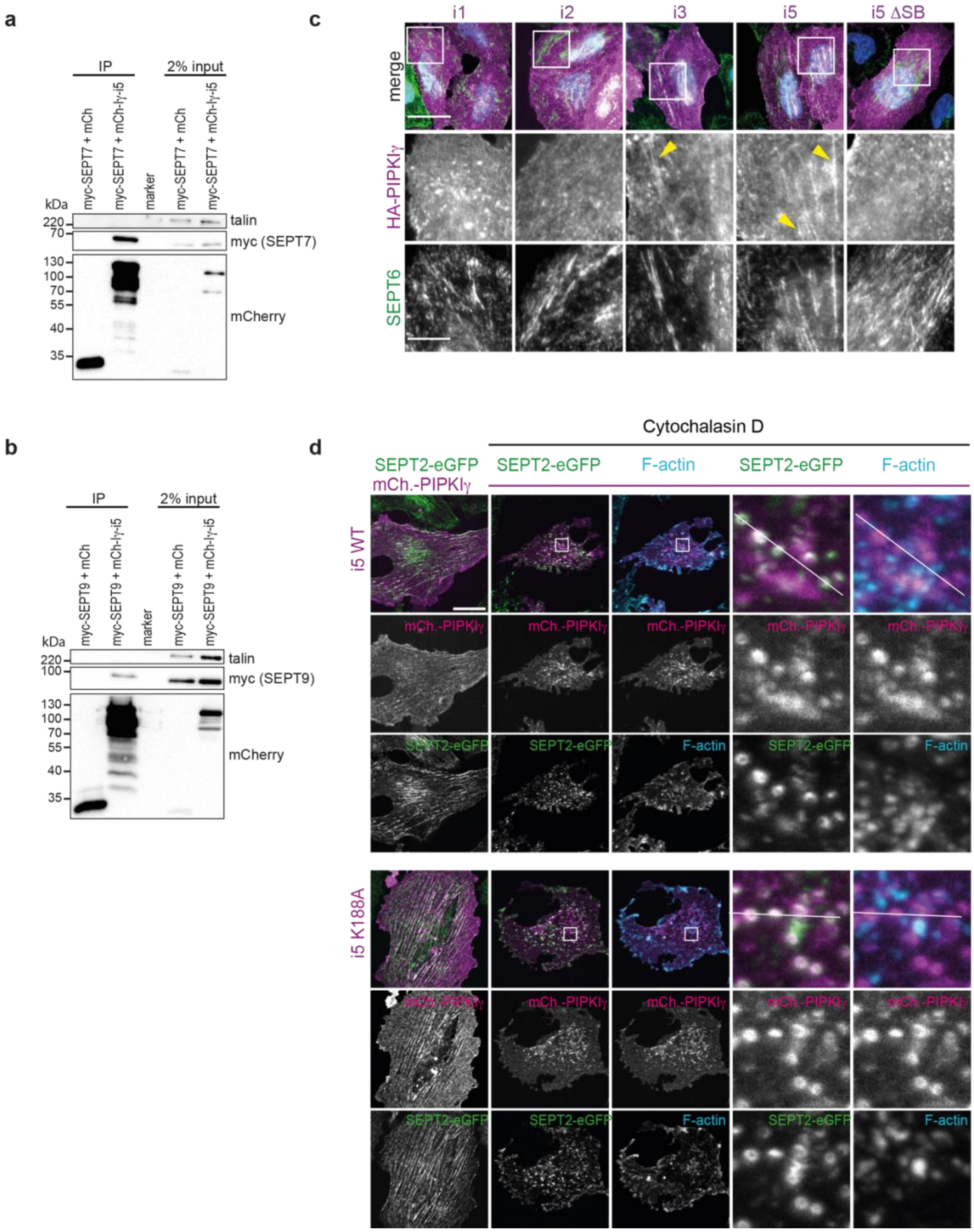

Supplementary Fig. 2 (related to Fig. 2) (a,b) Co-immunoprecipitation of myc-tagged SEPT7 **(a)** or SEPT9 **(b)** with mCherry-tagged PIPKIγ-i5 from HEK-293T cell lysates. HEK-293T cells were transiently cotransfected, and lysates were purified on an RFP-affinity resin. The retained material was analyzed by SDS-PAGE and Western blotting using the indicated antibodies. **(c)** Representative epi-fluorescence images derived from transfected HeLa cells. Over-expressed HA-tagged PIPKIγ-i3 or -i5, but not -i1, -

i2, or -i5ΔSB, exhibit a filamentous pattern (yellow arrowheads) overlapping with endogenous septin filaments (revealed by immunostaining of SEPT6) in HeLa cells. Scale bar, 30 µm (merge), and 10 µm (insets). **(d)** Single channel images of merges displayed in Fig. 2f. Source data are provided as a Source Data file.

Supplementary Figure 3

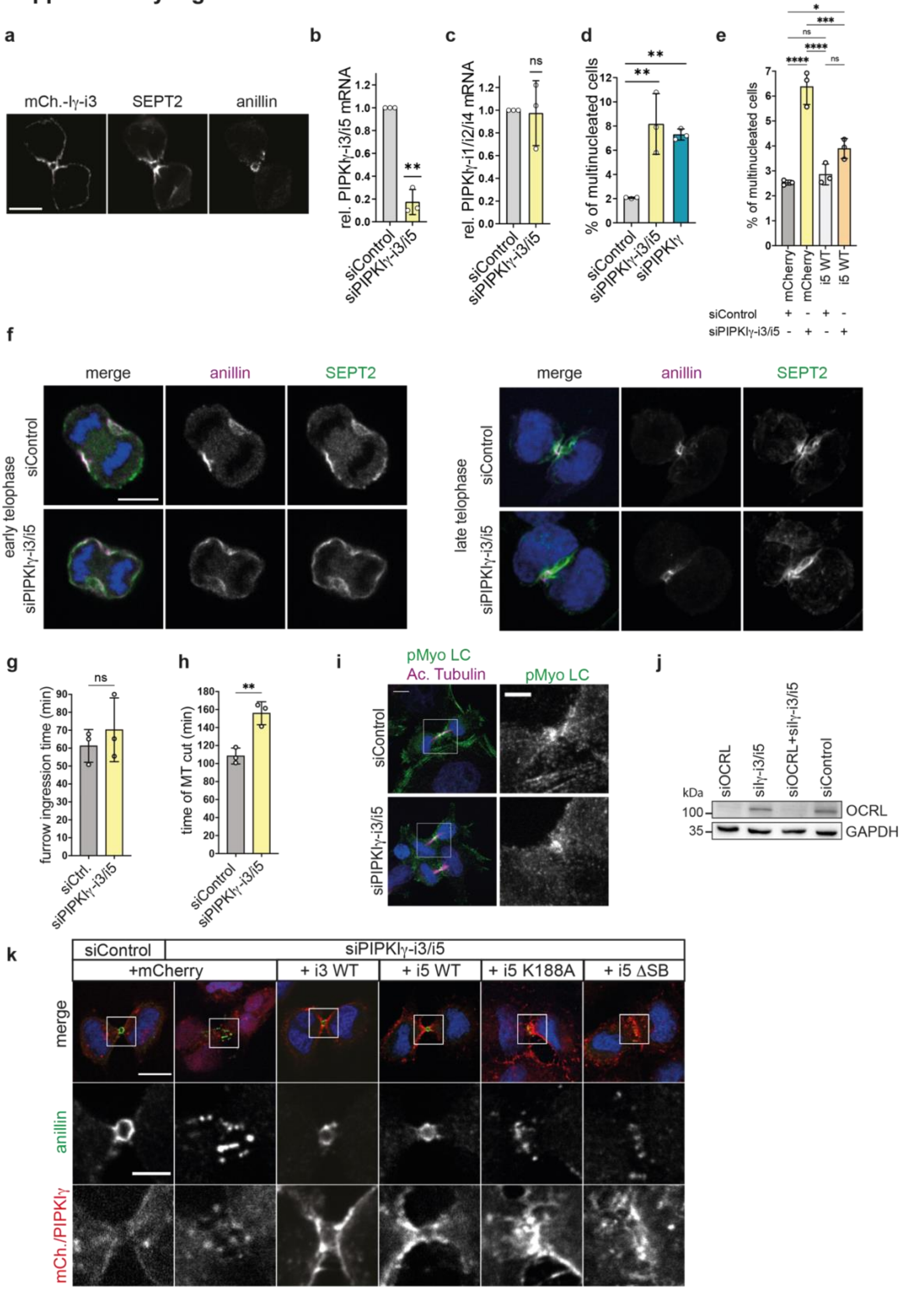

Supplementary Fig. 3 (related to Fig. 3) (a) Representative confocal images of HeLa cells stably expressing mCherry-PIPKIγ-i3, fixed at telophase and stained for anillin and SEPT2, scale bar: 10µm. **(b,c)** Knockdown of PIPKIγ-i3/i5 is specific and efficient. **(b)** Expression of PIPKIγ-i3+i5, normalized to GAPDH, and relative to control. **(c)** Expression of PIPKIγ-i1+i2+i4, normalized to GAPDH, and relative to control. Data are depicted as mean ± SD (n=3 independent experiments). Statistics: two-tailed, one sample t-test. (B). **(d)** Fraction of multinucleated cells upon treatment with isoform-specific siRNA, or with siRNA targeting all PIPKIγ isoforms. Data are depicted as mean ± SD (n=4 independent experiments). Statistics: 1-way ANOVA, followed by Dunnett's multiple comparison test. **(e)** Expression of siRNA-resistant mCherry-PIPKIγ-i5 (WT), but not of mCherry rescues multinucleation induced by treatment with siPIPKIγ-i3/i5. Data are depicted as mean ± SD (n=3 independent experiment). Statistics: 1-way ANOVA, followed by Tukey's multiple comparison test. **(f)** Representative confocal images of HeLa fixed at earlier stages of cytokinesis, and immunostained for anillin and SEPT2. Scale bar, 10µm. (*left*) Cells in early telophase, (*right*) cells in late telophase (i.e. upon complete ingression of the cleavage furrow, prior to ICB extension). **(g,h)** Live imaging of microtubule dynamics upon addition of SiR-tubulin to control or PIPKIγ-i3/i5-depleted cells. **(g)** Depletion of PIPKIγ-i3/i5 does not significantly impair furrow ingression, **(h)** but significantly delays timing of the microtubule cut. Data are depicted as mean ± SD (n=3 independent experiments). Statistics: two-tailed, unpaired t-test. **(i)** Representative confocal images of HeLa cells fixed at cytokinesis, and immunostained for phospho-myosin light chain (LC) and acetylated tubulin. Scale bar, 10µm (merge) and 5µm (insets). **(j)** Western blot analysis of lysates derived from HeLa cells treated with indicated siRNAs. **(k)** Representative confocal images derived from rescue experiments. Cells expressing mCherry or mCherry (mCh.)-tagged PIPKIγ-i3/i5 variants were treated with control or PIPKIγ-i3/i5-targeting siRNA. Synchronized cells fixed at late stage of cytokinesis immunostained for anillin. Scale bars, 15 µm (merge), 5µm (insets). *P < 0.05; **P < 0.01; ***P < 0.001; ****P < 0.0001. Source data and *P*-values are provided as a Source Data file.

Supplementary Figure 4

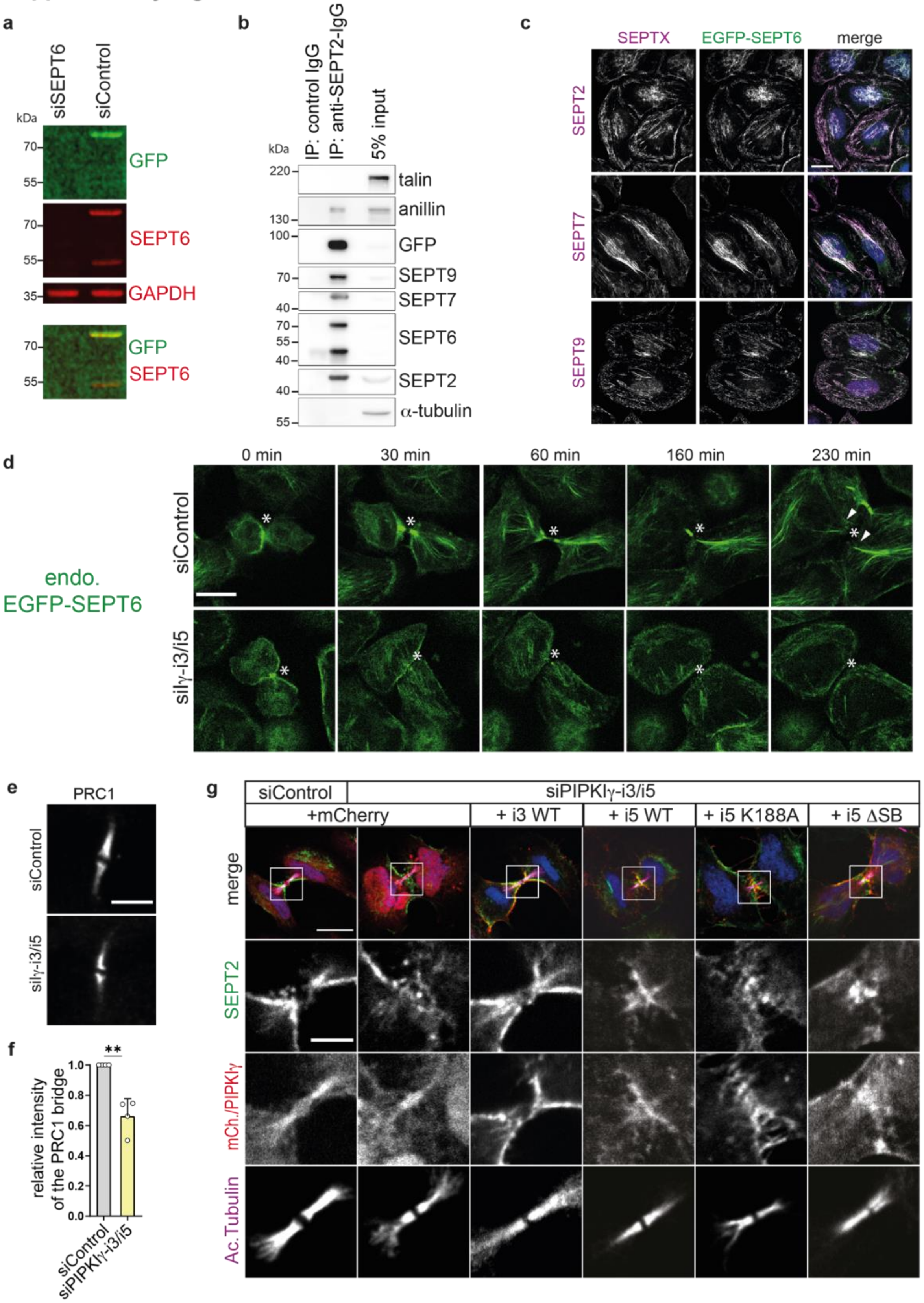

Supplementary Fig. 4 (related to Fig. 4) (a) Western blot analysis of lysates derived from genome-edited, eGFP-SEPT6-expressing HeLa cells treated with SEPT6-specific or control siRNAs. Lysates were separated by SDS-PAGE, and immunoblotted for GFP, SEPT6 and GAPDH. **(b)** Western blot analysis of SEPT2-immunoprecipitates obtained from lysates of genome-edited, eGFP-SEPT6 expressing HeLa cells. Precipitates were separated by SDS-PAGE, and immunoblotted for the indicated proteins. SEPT2 efficiently co-precipitates eGFP-SEPT6 and SEPT6, as well as other septin paralogs and anillin. **(c)** Representative confocal images derived from genome-edited, eGFP-SEPT6 expressing HeLa cells. Cells were fixed, and immunostained for endogenous SEPT2, SEPT7, or SEPT9. Scale bar, 10 µm. **(d)** Genome-edited HeLa cells expressing eGFP-SEPT6 cells were synchronized, and imaged throughout cytokinesis by confocal microscopy. Images show representative frames derived from movies of control- or PIPKIγ-i3/i5-depleted cells. Scale bar, 15µm. Asterisk indicates the putative midbody; arrowheads indicate sinuous septin fibers possibly deriving from the cytokinetic bridge. **(e)** Representative confocal images (max intensity z-projections) of PRC1 at the ICB, derived from control, or from PIPKIγ-i3/i5-depleted HeLa cells, upon synchronization at late stages of cytokinesis and immunostaining. Scale bar, 5 µm. **(f)** Relative intensity of PRC1. Normalized data are depicted as mean ± SD (n=4 independent experiments). Statistics: two-tailed one sample t test. **(g)** Representative confocal images derived from rescue experiments. Cells expressing mCherry or mCherry (mCh.)-tagged PIPKIγ-i3/i5 variants were treated with control or PIPKIγ-i3/i5-targeting siRNA. Synchronized cells were fixed at late stage of cytokinesis, and immunostained for acetylated tubulin and SEPT2. Scale bars, 15 µm (merge), 5 µm (insets). **P < 0.01; Source data and *P*-values are provided as a Source Data file.

Supplementary Figure 5

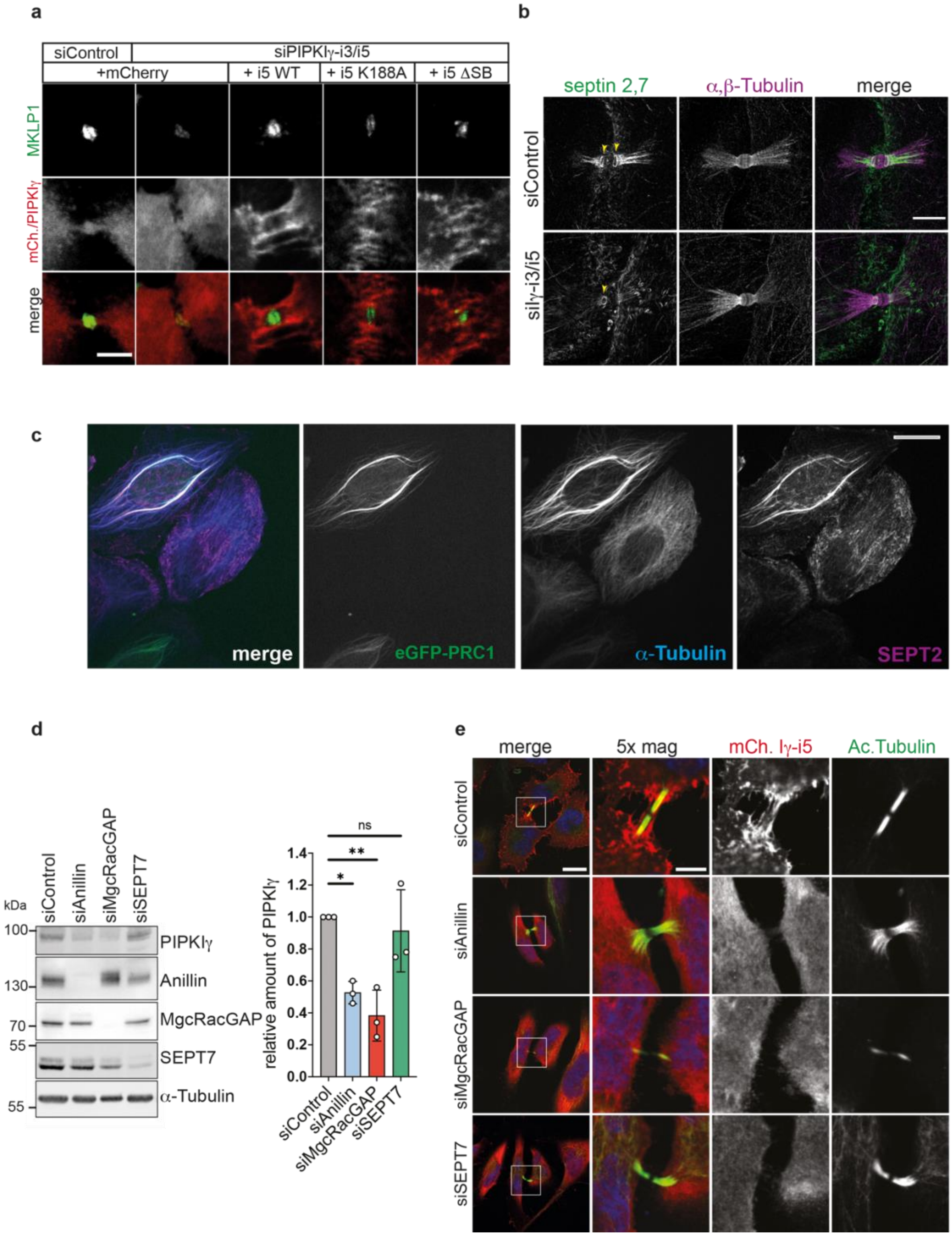

Supplementary Fig. 5 (related to Fig. 5) (a) Cells expressing mCherry or mCherry (mCh.)-tagged PIPKIγ-i3/i5 variants were treated with control or PIPKIγ-i3/i5-targeting siRNA, synchronized, fixed at late stage of cytokinesis, and immunostained for MKLP1. Scale bar, 5µm. **(b)** Representative confocal images derived from U-ExM (max-intensity projections of 21 slices with 1 µm spacing). Cells were immunostained for SEPT2/SEPT7 and α-/β-tubulin, and imaged on a spinning disk confocal microscope. Note that the two septins were stained simultaneously, and detected with the same secondary antibody to enhance the signal, akin to α-/β-tubulin. Scale bar, 10µm. **(c)** Representative images of HeLa cells transiently expressing eGFP-PRC1, immunostained upon fixation for α-tubulin and SEPT2. **(d)** Depletion of anillin or of MgcRacGAP, but not of SEPT7 reduces global levels of PIPKIγ. (*left*) Lysates derived from HeLa cells treated with the indicated siRNAs were separated by SDS-PAGE and analyzed by immunoblotting using the indicated antibodies. (*right*) Quantitative analysis of PIPKIγ expression levels as detected by fluorescently labeled secondary antibodies (n=3 independent experiments). Statistics: 1-way ANOVA, followed by Dunnett's multiple comparison test. **(e)** Representative images of HeLa cells stably expressing mCherry-PIPKIγ-i5 (WT) treated with indicated siRNAs. Cells were fixed at late stages of cytokinesis and immunostained for mCherry and acetylated tubulin. Scale bar 20µm (merge) and 5µm (insets). *P < 0.05; **P < 0.01. Source data and *P*-values are provided as a Source Data file.
