# Supplementary material for: Local PI(4,5)P2 synthesis by septin-associated PIPKIγ isoforms controls centralspindlin association with the midbody during cytokinesis
Source: Nat Commun. 2026 Feb 7;17:1482. doi: 10.1038/s41467-026-69224-3 (PMC12886786; doi:10.1038/s41467-026-69224-3)
Supplement: Supplementary file 2 — Description of Additional Supplementary Files [file 41467_2026_69224_MOESM2_ESM.pdf]

<u>Description of Additional Supplementary Files</u>

Supplementary Movie 1: Dynamic rearrangement of eGFP-SEPT6 during cell division in control cells.
HeLa eGFP-SEPT6 knock-in cells were treated with control siRNA, synchronized, and imaged throughout cytokinesis with a confocal microscope. Imaging was started 7,5h after thymidine release, and carried out with a frame rate of 10 minutes. Time stamp: hh:mm; scale bar, 15µm.

Supplementary Movie 2: Dynamic rearrangement of eGFP-SEPT6 during cell division in PIPKI[illegible]-i3/i5-depleted cells.
HeLa eGFP-SEPT6 knock-in cells were treated with siRNA targeting PIPKI[illegible]-i3/i5, synchronized, and imaged throughout cytokinesis with confocal microscope. Imaging was started 7,5h after thymidine release, and carried out with a frame rate of 10 minutes. Time stamp: hh:mm; scale bar, 15µm.
