# Supplementary material for: Local PI(4,5)P2 synthesis by septin-associated PIPKIγ isoforms controls centralspindlin association with the midbody during cytokinesis
Source: Nat Commun. 2026 Feb 7;17:1482. doi: 10.1038/s41467-026-69224-3 (PMC12886786; doi:10.1038/s41467-026-69224-3)
Supplement: Supplementary file 5 — Reporting Summary [file 41467_2026_69224_MOESM5_ESM.pdf]

nature portfolio

Corresponding author(s): Volker Haucke, Michael Krauss

Last updated by author(s): Dec 7, 2025

Reporting Summary

Nature Portfolio wishes to improve the reproducibility of the work that we publish. This form provides structure for consistency and transparency in reporting. For further information on Nature Portfolio policies, see our Editorial Policies and the Editorial Policy Checklist.

Statistics

For all statistical analyses, confirm that the following items are present in the figure legend, table legend, main text, or Methods section.

n/a	Confirmed	
☐	☒	The exact sample size (*n*) for each experimental group/condition, given as a discrete number and unit of measurement
☐	☒	A statement on whether measurements were taken from distinct samples or whether the same sample was measured repeatedly
☐	☒	The statistical test(s) used AND whether they are one- or two-sided<br>*Only common tests should be described solely by name; describe more complex techniques in the Methods section.*
☒	☐	A description of all covariates tested
☐	☒	A description of any assumptions or corrections, such as tests of normality and adjustment for multiple comparisons
☐	☒	A full description of the statistical parameters including central tendency (e.g. means) or other basic estimates (e.g. regression coefficient) AND variation (e.g. standard deviation) or associated estimates of uncertainty (e.g. confidence intervals)
☐	☒	For null hypothesis testing, the test statistic (e.g. *F*, *t*, *r*) with confidence intervals, effect sizes, degrees of freedom and *P* value noted<br>*Give P values as exact values whenever suitable.*
☒	☐	For Bayesian analysis, information on the choice of priors and Markov chain Monte Carlo settings
☒	☐	For hierarchical and complex designs, identification of the appropriate level for tests and full reporting of outcomes
☐	☒	Estimates of effect sizes (e.g. Cohen's *d*, Pearson's *r*), indicating how they were calculated

Our web collection on statistics for biologists contains articles on many of the points above.

Software and code

Policy information about availability of computer code

Data collection	Microscopy data was routinely collected with a Zeiss confocal spinning disk microscope (Yokogawa CSU22, Hamamatsu EMCCD camera) under control of Volocity software (Perkin Elmer). Alternatively, imaging was conducted with a Nikon Eclipse Ti microscope (illumination: CoolLED, pE4000, prime95B sCMOS camera) operated by NIS-Elements software, or with an Olympus spinning disk microscope (Yokogawa CSU-X1, Hamamatsu C11440 camera). Western blot analysis and image processing was carried out on a Biorad ChemiDoc Imaging System, controlled by Image Lab software, version 6.1.0, build 7, or by LICORbio controlled by ImageStudioLite software.
Data analysis	Immunofluorescence images were processed using FIJI Version : 2.1.0/1.53c. All statistical tests were performed using Graphpad Prism9 or 10. Wester blot analyses were performed with Image Lab or ImageStudioLite.

For manuscripts utilizing custom algorithms or software that are central to the research but not yet described in published literature, software must be made available to editors and reviewers. We strongly encourage code deposition in a community repository (e.g. GitHub). See the Nature Portfolio guidelines for submitting code & software for further information.

Data

Policy information about availability of data

All manuscripts must include a data availability statement. This statement should provide the following information, where applicable:

- Accession codes, unique identifiers, or web links for publicly available datasets
- A description of any restrictions on data availability
- For clinical datasets or third party data, please ensure that the statement adheres to our policy

The authors declare that all all relevant information supporting the findings of this study are available within the paper and its supplementary files, or are available from the corresponding authors. Source Data are provided with this paper.

Research involving human participants, their data, or biological material

Policy information about studies with human participants or human data. See also policy information about sex, gender (identity/presentation), and sexual orientation and race, ethnicity and racism.

Reporting on sex and gender	N/A
Reporting on race, ethnicity, or other socially relevant groupings	N/A
Population characteristics	N/A
Recruitment	N/A
Ethics oversight	N/A

Note that full information on the approval of the study protocol must also be provided in the manuscript.

Field-specific reporting

Please select the one below that is the best fit for your research. If you are not sure, read the appropriate sections before making your selection.

☒ Life sciences ☐ Behavioural & social sciences ☐ Ecological, evolutionary & environmental sciences

For a reference copy of the document with all sections, see nature.com/documents/nr-reporting-summary-flat.pdf

Life sciences study design

All studies must disclose on these points even when the disclosure is negative.

Sample size	No sample size calculations were performed, instead sample sizes were empirically determined based on the experimenter's estimation of variability.
Data exclusions	Data points were excluded based on predefined criteria determined by the experimenter: Knockdown efficiency, cell viability (e.g. exclusion of blebbing cells)
Replication	Most of experimental findings were replicated over at least 3 independent experiments. Number of replicates is indicated in figure legends.
Randomization	N/A
Blinding	Immunofluorescence images were captured blindly by selecting cells in the Hoechst/DAPI channel, or by applying by applying fully automated analysis procedures.

Reporting for specific materials, systems and methods

We require information from authors about some types of materials, experimental systems and methods used in many studies. Here, indicate whether each material, system or method listed is relevant to your study. If you are not sure if a list item applies to your research, read the appropriate section before selecting a response.

Materials & experimental systems

n/a	Involved in the study
☐	☒ Antibodies
☐	☒ Eukaryotic cell lines
☒	☐ Palaeontology and archaeology
☒	☐ Animals and other organisms
☒	☐ Clinical data
☒	☐ Dual use research of concern
☒	☐ Plants

Methods

n/a	Involved in the study
☒	☐ ChIP-seq
☒	☐ Flow cytometry
☒	☐ MRI-based neuroimaging

Antibodies

Antibodies used

Acetylated tubulin (mouse) Sigma-Aldrich T7451; α-tubulin (mouse) Sigma-Aldrich T5168; β-tubulin (mouse) Sigma-Aldrich T5293; PIPKIa(mouse) Santa-Cruz sc-398687; PIPKIb(mouse) Santa-Cruz sc-514169; PIPKIc(rabbit) Home-made in this study ; SEPT2 (rabbit) Sigma-Aldrich HPA018481; SEPT5 (mouse) Santa-Cruz sc-20040; SEPT6 (rabbit) Home-made in this study ; SEPT7 (rabbit) Santa-Cruz sc-20620; SEPT7 (rabbit) TECAN JP18991; SEPT3 (mouse) Sigma-Aldrich WH0055964M3; SEPT9 (rabbit) (Diesenberg et al., 2015); SEPT9 (mouse) abnova H0001 1081-M01; Talin (mouse) Sigma-Aldrich T3287; GAPDH (mouse) Sigma-Aldrich G8795; Anillin (goat) Abcam ab5910; c-Myc (mouse) Hybridoma clone obtained from DSHB, purified ourselves 9E10; RFP (rabbit) Clontech 632496; HA (mouse) Abcam ab18181; OCRL1 (rabbit) Cell Signaling 8797; PRC1 (mouse) Thermo-Fisher MA1-846; CIT-K (mouse) BD Transduction Laboratories 611377; CIT-K (rabbit) Abcam ab86782; MKLP1 (rabbit) GeneTex GTX120875; MgcRacGAP (goat) Abcam Ab2270 MgcRacGAP (rabbit) Proteintech 13739-1-AP; GFP (mouse) Clontech 632381; GFP (rabbit) Abcam ab6556; PI(4,5)P2 (mouse, IgM) Echelon Biosciences Z-P045; pMyosin LC (ser 19) Cell signaling 3671

Validation

Commercially available antibodies were selected based on previous publications, or on information provided by manufacturers. Antibodies generated in our own lab are routinely validated by knockdown experiments (SEPT6: Supplementary Figure 4a; PIPKIc: Supplementary Figure 1a; SEPT9: Diesenberg et al., 2015).

Eukaryotic cell lines

Policy information about cell lines and Sex and Gender in Research

Cell line source(s)

HeLa(ATCC, #CCL-2), HEK 293T (ATCC, #CRL-3216), genome-engineered NRK49F SEPT2-eGFP (Banko et al., Cytokeleton, 2019)

Authentication

Cell lines from ATCC are regularly authenticated by STR profiling and were used by us without further authentication.

Mycoplasma contamination

Cell lines were regularly tested for mycoplasma contamination via Mycoplasma detection PCR kit or visible contamination of nuclei stain, and no contamination was observed.

Commonly misidentified lines
(See ICLAC register)

No commonly misidentified cell lines were used.

Plants

Seed stocks

N/A

Novel plant genotypes

N/A

Authentication

N/A
